# Verteporfin Is a Promising Anti-Tumor Agent for Cervical Carcinoma by Targeting Endoplasmic Reticulum Stress Pathway

**DOI:** 10.3389/fonc.2020.01781

**Published:** 2020-09-03

**Authors:** Meng Wang, Chang Liu, Yuehan Li, Qiulin Zhang, Lixia Zhu, Zishui Fang, Lei Jin

**Affiliations:** ^1^Reproductive Medicine Center, Tongji Hospital, Tongji Medical College, Huazhong University of Science and Technology, Wuhan, China; ^2^Department of Reproductive Medicine, Maternal and Child Health Hospital of Hubei Province, Tongji Medical College, Huazhong University of Science and Technology, Wuhan, China

**Keywords:** cervical cancer, verteporfin, apoptosis, endoplasmic reticulum stress, fertility preservation

## Abstract

Accumulated evidence has shown that the photosensitizer Verteporfin (VP) may be an ideal agent for various cancer types. However, the effect and mechanism of VP on human cervical carcinoma remain rudimentary. The aim of this study was to investigate the effect of VP on human cervical carcinoma cells (HeLa and SiHa cells) and to elucidate the possible mechanism. CCK-8, wound healing assay, flow cytometry analysis, western blotting, TUNEL staining were performed to evaluate the effects of VP on HeLa and SiHa cells *in vitro* as well as *in vivo* on a xenograft model. In addition, the role of endoplasmic reticulum (ER) stress in VP-induced apoptosis was investigated using RT-qPCR and western blotting. The results showed that the viability of HeLa and SiHa cells was suppressed by VP in dose- and time-dependent manners. Compared with the control group, apoptosis rates were higher with stronger TUNEL fluorescence signals in the experimental group, which substantiated that VP induced apoptosis at both 2D and 3D cell levels. Besides, VP can squelch the growth of tumors in both sizes and weights on the xenograft models without impairing ovarian reserve. Mechanism studies demonstrated that VP activated ER stress by upregulating the expression of GRP78, CHOP, and Caspase-12, and VP-induced apoptosis can be alleviated when ER stress pathway was inhibited. Our results provided a foundation for repurposing VP as a promising agent for cervical cancer patients without obvious reproductive toxicity by targeting ER stress pathway, and more researches are required to support its application in clinical practice.

## Introduction

An annual mortality of 266,000 with 528,000 incidence cases worldwide made cervical cancer the most common carcinoma in female reproductive system (1). The data showed that approximately 90% of cervical cancer deaths were from developing countries (2), and the incidence rate of cervical cancer in China, the biggest developing country, has showed an upward and younger trend (3). Currently, cisplatin-based sequential chemo-radiation therapy is the standard approach for patients in FIGO stage IIB to IVA (4). However, various studies have indicated that the development of resistance to cisplatin substantially compromised the efficacy of cisplatin to treat advanced or recurrent cervical cancer (5). Moreover, cisplatin also induces severe reproductive toxicity and genotoxicity in mammals (6). Therefore, the existing chemotherapy strategy is quite inappropriate for cervical cancer patients with imperative procreation desire. It is necessary to find a new type of chemotherapy agent with less reproductive toxicity on cervical cancer.

Photodynamic therapy (PDT) is a photosensitizer-based non-invasive novel therapy, utilized in the treatment of various diseases including tumors of assorted types (7). The photosensitizer was targeted in pathological tissues and activated by light of a specific wavelength, inducing selective cell death, but sparing the surrounding normal tissues (8). Verteporfin (VP), a hydrophobic photosensitizer, can combine with low density lipoprotein (LDL) and be transferred into cytoplasm through LDL receptors on proliferative cells such as malignant cells (9). Recently, some studies reported that VP could still induce apoptosis in cancer cells without light activation (10). It can also inhibit interaction of the key components in Hippo pathway, which is related to cell growth, proliferation and differentiation (11). In addition, continuous injection of VP into mammals showed no negative effect on the reproductive systems (12). Therefore, VP is a potential anti-tumor agent without obvious reproductive toxicity, although the exact mechanism remains unclear.

Endoplasmic reticulum (ER) stress is a condition that unfolded or misfolded proteins anomalously accumulate in cytoplasm, leading to ER homeostasis restoring or cell self-destruction, which depends on the intensity and duration of stressor (13). ER stress, which can be caused by any substances affecting cell homeostasis, is considered to play a vital role in the occurrence and development of various diseases (14). Thus, we speculated that VP might induce apoptosis in tumor cells via ER stress pathway, resulting in anti-tumor effect on human cervical carcinoma.

This study intended to investigate the therapeutic effect of VP on human cervical carcinoma and to explore its possible mechanism to provide theoretical evidence for the promotion of the photosensitizer represented by VP for oncological diseases treatment in the clinic.

## Materials and Methods

### Agents and Cell Culture

Human cervical cancer cell lines HeLa and SiHa were gifts from Cancer Biology Research Center of Tongji Hospital, Tongji Medical College, Huazhong University of Science and Technology. Cells were cultured in DMEM/F12 medium (Hyclone, Logan, UT, United States) supplemented with 10% (*v/v*) fetal bovine serum (FBS, Gibco, Carlsbad, CA, United States), 100 U/mL penicillin G and 100 μg/mL streptomycin (Boster, Wuhan, China) in a 37°C incubator with 5% CO_2_.

Phosphate buffer saline (PBS) and Trypsin-EDTA solution were purchased from Promoter (Wuhan, China). VP (S1786, Selleck, Shanghai, China) was dissolved in DMSO (Promoter, Wuhan, China) to prepare the stock solution at a concentration of 10 mM, as the manufacturer’s instructions recommended. Then, the stock solution was diluted to suitable concentrations by the medium in different experimental groups. Thapsigargin (TG) (T9033, Sigma, St. Louis, MO, United States) of 1 μM was used to induce ER stress as a positive control. Cells were pretreated with 5 mM 4-phenyl butyric acid (4-PBA, an ER stress inhibitor) (S4125, Selleck, Shanghai, China) dissolved in PBS for 2 h to inhibit ER stress. The following primary antibodies were diluted at a ratio of 1:1000 unless stated otherwise. Anti-GRP78 and anti-CHOP were purchased from CST (Danvers, MA, United States); anti-Cleaved Caspase-3 from R&D (United States); anti-Bcl-2, anti-Bax (1:2000) and anti-Caspase-12 from Proteintech (Wuhan, China); and anti-β-actin from ABclonal (Wuhan, China). HRP-conjugated secondary antibodies (1:2000) were purchased from Servicebio (Wuhan, China). Each experiment was performed for three times independently.

### Cell Viability Assay

The viability of cells was detected by CCK-8 kit (MCE, Shanghai, China). HeLa and SiHa cells were suspended in the serum-free medium and seeded in the 96-well plates at a density of 5000 cells per well overnight for cells to attach. Various concentrations (0.5, 1.0, 5.0, and 10.0 μM) of VP or DMSO (vehicle control group) was added into the medium and incubated with cells for 0 h, 24 h, 48 h, and 72 h at 37°C with 5% CO_2_. Then, 10 μL CCK-8 reagent was added into each chamber, and incubated for 2 h under the same condition. Cell viability was obtained at the absorbance of 450 nm using a spectrophotometer (BioTek Instruments, Inc., Winooski, VT, United States).

### Wound Healing Assay

After overspreading the chambers in 6-well plates, well-attached HeLa and SiHa cells were scratched by a pipette tip to generate a scratch wound after experiencing serum starvation for 12 h. Then, the confluent cells were washed in sterile PBS for three times to remove the debris and replenished with serum-free medium. In the experiment group, cells were incubated with 1 μM VP for 48 h. The wound areas were measured every 24 h using a microscope (Axio Observer A1, Carl Zeiss, Germany). The percentages of wound closure were calculated by ImageJ software (NIH, Bethesda, MD, United States).

### Flow Cytometry Analysis

The rate of apoptosis was measured by flow cytometry using Annexin V-FITC/PI kit (Cat. No. 556547, BD Biosciences, United States). Cells were pre-treated with 1 μM VP for 12 h, 24 h, 36 h, 48 h, respectively, and 0.1% DMSO (*v/v*) as control group. Pre-treated cells were washed and re-suspended using 1× Binding buffer (1 mL 10 × Binding buffer in 9 mL ddH_2_O), and subsequently stained with 5 μL Annexin V-FITC as well as 5 μL PI for 20 min away from light at room temperature. Apoptotic cells were analyzed by flow cytometry (BD FACSCalibur Cell Sorting System, United States).

### Reverse Transcription-Quantitative PCR (RT-qPCR) Analysis

Cells were treated as mentioned above. NucleoZol reagent (Macherey-Nagel, Germany) was used for total RNA extraction from the cells. Then, 500 ng isolated RNA was immediately converted into cDNA using cDNA synthesis kit (RR036A, Takara, Tokyo, Japan) according to manufacturer’s instructions. The qPCR reaction mixture contained 10 μL TB Green *Premix Ex Taq* (RR420A, Takara, Tokyo, Japan), 1 μL PCR Forward Primer (10 μM), 1 μL PCR Reverse Primer (10 μM), 1 μL prepared cDNA and 7 μL RNase-free water. qPCR was performed in a condition including 40 cycles of denaturation at 95°C for 5 s, annealing at 60°C for 20 s, and extension at 72°C for 30 s. The sequences of the primers utilized were designed as follows: GRP78: 5′-GACGCTGGAACTATTGCTGGC-3′ (F), 5′-AGCTGCCGT AGGCTCGTT-3′ (R), CHOP: 5′- GCACCTC CCAGAGCCCTCACTCTCC-3′ (F), 5′-GTCTACTCCAAGCC TTCCCCCTGCG-3′ (R) and β-actin: 5′- TGACGTGGACAT CCGCAAAG-3′ (F), 5′-CTGGAAGGTGGACAGCGAGG-3′ (R). The relative quantification of RNA expression level was calculated by using the 2^–Δ^ ^Δ^ ^*ct*^ method and normalized to β-actin.

### Western Blotting Analysis

Endoplasmic reticulum stress was induced by the incubation with 1 μM TG for 48 h in positive control. Cells after treatment were collected and total proteins were isolated by RIPA lysis buffer (Servicebio, Wuhan, China) with 2% Protease inhibitor cocktail (MCE, Shanghai, China). The concentration of protein was determined by BCA assay (Vazyme, Nanjing, China). The proteins were separated by 10% SDS-PAGE and then transferred to 0.45 μm PVDF membranes (Millipore, Billerica, MA, United States). Subsequently, the membranes were blocked by 5% skim milk (Servicebio, Wuhan, China) for 1 h at 37°C and incubated with primary antibodies at 4°C overnight. Before being detected by ECL kit (HY-K1005, MCE, China), the membranes were incubated with corresponding secondary antibodies for 1 h in a 37°C shaker. The protein expression level was quantified by densitometry with the use of ImageJ software (NIH, Bethesda, MD, United States).

### TUNEL Assay of 3D Cell Model

The 3D cell models of HeLa and SiHa cells were established in Matrigel Basement Membrane Matrix (BD, Franklin Lakes, NJ, United States). Cell suspensions were mixed with Matrigel, and 300 μL mixture was added into per chamber in 24-well culture plate, which was pre-coated with Matrigel according to manufacturer’s instructions. Each well contained 5 × 10^5^/ml cells with 90% (*v/v*) Matrigel and then incubated for 30 min at 37°C before another 500 μL culture medium added into the chambers. The medium was replaced every 2 days, and the cells were cultured for 10 days until they gathered and formed spheres. After treatment with 1 μM VP or DMSO (vehicle control) for 48 h, 3D cells in the Matrigel were washed and fixed in 4% paraformaldehyde for 1 h at room temperature. Samples were incubated with reaction mixtures in TUNEL kit (Roche, Shanghai, China) in dark for 1 h and were stained with DAPI for 5 min. The images were captured using a fluorescence microscope (Axio Observer A1, Carl Zeiss, Germany).

### Tumorigenicity Assay

The tumorigenicity assay of nude mice was approved by the Ethics Committee of Tongji Hospital, Tongji Medical College, Huazhong University of Science and Technology. Cervical squamous carcinoma is most common cervical carcinoma type in clinical, thus we established xenograft models using SiHa cells. Three-week-old female BALB/c nude mice (*n* = 5 per group) were purchased from Charles River, Beijing, China. The animals were kept on 12 h light/12 h darkness regimen and free to food and water in the SPF Animal Laboratory of Tongji Hospital. After 1-week suiting period, the mice were subcutaneously injected with 1 × 10^7^ SiHa cells suspended in 100 μL PBS. Tumor growth was measured every day, and when the diameters of tumors reached 0.5 cm, the mice were randomly divided into two groups. In the experimental group, the animals were intraperitoneally injected with 100 mg/kg VP (dissolved in DMSO and diluted by normal saline) every 3 days for five times as previously described (15), and the animals in control group received the equal volume of vehicles (DMSO in normal saline). The mice were sacrificed 2 days after the last injection, and the three mutually orthogonal diameters (d1, d2, d3) as well as the weights of the tumors were measured. The tumor volumes were determined according to the formula: $V=\frac{\pi }{6}\,d\,1\,d\,2\,d\,3$.

### Morphological Classification of Follicles

After VP or vehicle treatment, ovaries xenograft mice were collected, and the all left ovaries were fixed in 4% paraformaldehyde (Servicebio, Wuhan, China) overnight and then embedded in paraffin wax. The paraffin-embedded ovaries were sectioned into 4-μm slides completely, followed by the stain of hematoxylin and eosin (H&E) (Biosci, Wuhan, China). The number of follicles in every 10th serial section was counted using a light microscope. The follicle categories were classified according to the morphology of oocytes and granulosa cells as previously described (16). Briefly, follicles were defined as primary if the oocyte was surrounded by a single layer of cuboidal granulosa cells. Secondary follicles possessed an oocyte surrounded by more than one layer of cuboidal granulosa cells without visible antrum. Antral follicles were exhibited as a defined cumulus granulosa cell layer with a clearly antral space. The zona pellucida and oocyte in atresia follicles were deformed and readily recognized. The total number of follicles in every 10th serial section was summed and the percentage of each category was calculated.

### Serum Anti-müllerian Hormone Measurement

Following VP or vehicle treatment, the whole blood of xenograft mice was collected via retro-orbital bleeding. Mouse anti-müllerian hormone (AMH) ELISA kit (CSB-E13156m, CUSABIO, Wuhan, China) was used to measure serum AMH level. Whole blood was rest at room temperature for 1 h, and then centrifuged for 30 min at 1000 *g* at 4°C to isolate serum. A mixture of 50 μL standard or sample and 50 μL HRP-conjugate mixed solution was added into each well. After incubating for 1 h at 37°C, each well was washed three times using 200 μL Wash Buffer. Then 50 μL of Substrate A and 50 μL of Substrate B were added to each well for 15 min incubation in darkness at 37°C prior to the additional 50 μL Stop Solution. The optical density of each wells was determined within 10 min at 450 nm using a spectrophotometer (BioTek Instruments, Inc., Winooski, VT, United States).

### Statistical Analysis

Statistical analyses were performed by using Statistical Product and Service Solutions 22.0 (SPSS, IBM, Armonk, NY, United States). Experimental results were presented as the mean ± standard deviation (SD). Student’s independent *t*-test were used for statistical comparisons between two groups. Two-tailed hypothesis tests were performed. *P* < 0.05 was considered as significantly statistical differences.

## Results

### VP Suppressed Cell Viability and Cell Migration *in vitro*

**FIGURE 1:**
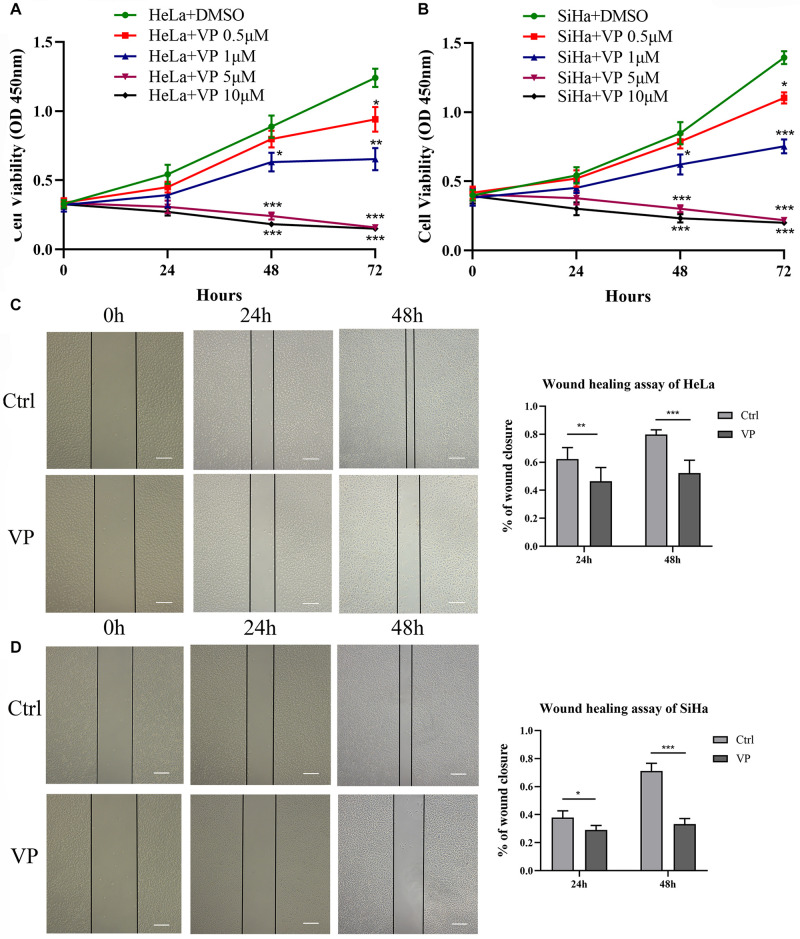
VP suppressed cell viability and migration in human cervical cancer cells. HeLa **(A)** and SiHa **(B)** cells were treated with 0.5 μM, 1.0 μM, 5.0 μM, 10.0 μM VP or DMSO (vehicle control group), respectively. Cell viability was assessed by CCK-8 assay. Scratched HeLa **(C)** and SiHa **(D)** cells were incubated with DMSO or 1 μM VP for 48 h. The percentage of wound closure was calculated every 24 h. Data were presented as mean ± SD. Scale bar = 200 μm. Each experiment was performed for three times independently. Data were significantly different from respective control group (*P < 0.05; **P < 0.01; ***P < 0.001). VP, verteporfin; DMSO, dimethyl sulfoxide.

CCK-8 assay was performed to evaluate the effect of VP on the viability of cervical carcinoma cells. HeLa and SiHa cells were treated with various concentrations of VP (0.5, 1.0, 5.0, 10.0 μM) or DMSO (vehicle control) for 0 h, 24 h, 48 h, and 72 h. As shown in Figures 1A,B, cell viability was inhibited by VP in dose- and time-dependent manners. Based on the CCK-8 viability assay, 1.0 μM concentration was chosen for the subsequent experiments.

The effect of VP on the cell migration was assessed by wound healing assay. Scratched HeLa and SiHa cells were treated with 0.1% DMSO (*v/v*) or 1.0 μM VP for 48 h, and images were captured every 24 h. The results showed that compared with the control group, the proportion of wound closures in the VP group was significantly decreased in both 24 h and 48 h (Figures 1C,D), which indicated that VP could significantly inhibit the migration of human cervical carcinoma cells.

### VP Efficiently Induced Cell Apoptosis *in vitro*

**FIGURE 2:**
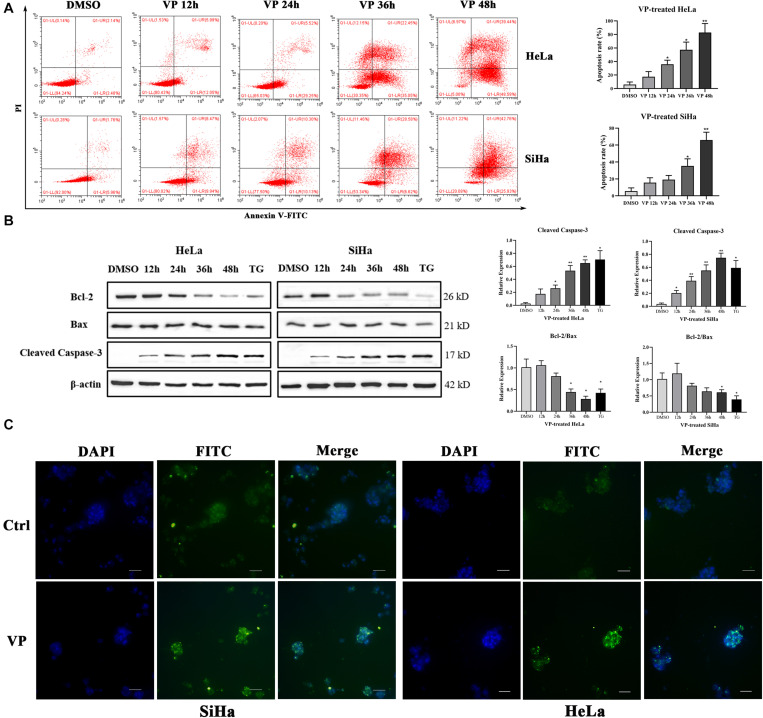
VP induced apoptosis in human cervical cancer cells *in vitro*. HeLa and SiHa cells were incubated with 1 μM VP for 12 h, 24 h, 36 h and 48 h, DMSO (vehicle control group) or 1 μM TG (positive control group) for 48 h. **(A)** Apoptosis was detected using flow cytometry. **(B)** Apoptosis-related proteins were investigated by western blotting in the VP-treated HeLa and SiHa cells. **(C)** Matrigel-embedded 3D HeLa and SiHa cells were incubated with DMSO (vehicle control) or 1 μM VP for 48 h, then, the apoptosis was detected by TUNEL staining kit and the representative images were captured using a fluorescence microscope. Data were presented as mean ± SD. Scale bar = 100 μm. Each experiment was performed three times independently. Data were significantly different from respective control group (*P < 0.05; **P < 0.01). DMSO, dimethyl sulfoxide; VP, verteporfin; DAPI, diamidino-2-phenylindole; Ctrl, control group.

Apoptosis induced by VP was investigated in human cervical carcinoma cells at both 2D and 3D cell levels. HeLa and SiHa cells were treated with 1.0 μM VP for 0 h, 12 h, 24 h, 36 h, and 48 h, respectively. Then, the percentage of apoptotic cells was assessed by Annexin V-FITC/PI flow cytometry analysis. Signals can be detected in PI channels in all VP-treated groups because of the photosensitivity of colored VP, apoptotic cells were, therefore, referred to only FITC-positive cells. It was obvious that the apoptosis rates increased dramatically in a time-dependent manner in both cell types (Figure 2A).

Subsequently, the protein expression levels of Cleaved Caspase-3, Bcl-2, and Bax, which were critical apoptosis-related molecules, were measured by western blotting. The results showed that the expression of Cleaved Caspase-3 was significantly upregulated and the ratio of Bcl-2 and Bax was decreased with the extension of VP exposure time (Figure 2B). These data indicated that VP was able to induce cell apoptosis in the 2D level. In addition, Matrigel-embedded 3D models of SiHa cells were established. The results showed that the intensity of TUNEL fluorescence signals was higher in the experimental group in both HeLa and SiHa cells (Figure 2C), which indicated that cell apoptosis can be induced by VP at 3D cell level.

### VP Activated ER Stress Pathway *in vitro*

**FIGURE 3:**
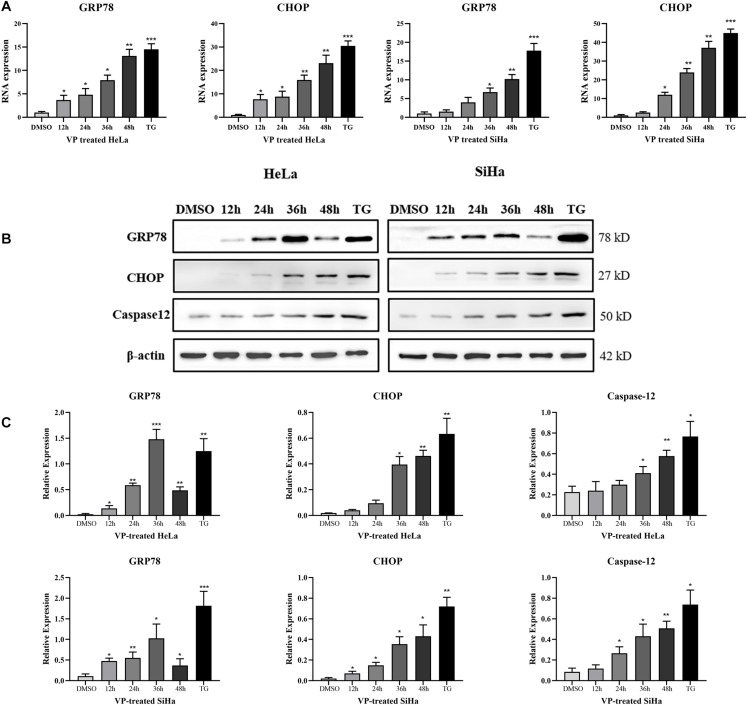
VP activated ER Stress in human cervical cancer cells *in vitro*. HeLa and SiHa cells were incubated with 1 μM VP for 12 h, 24 h, 36 h, and 48 h, DMSO (vehicle control group) or 1 μM TG (positive control group) for 48 h. **(A)** RNA levels of GRP78 and CHOP were assessed by RT-qPCR. **(B,C)** Protein expression of GRP78, CHOP, and Caspase-12 was assessed by western blotting. Data were presented as mean ± SD. Each experiment was performed for three times independently. Data were significantly different from the control (DMSO) group (*P < 0.05; **P < 0.01; ***P < 0.001). DMSO, dimethyl sulfoxide; VP, verteporfin.

To explore the mechanism of human cervical carcinoma cell apoptosis induced by VP, the RNA and protein expression levels of several key molecules of ER stress pathway were measured by RT-qPCR and western blotting. The results showed that VP can up-regulate the RNA (Figure 3A) and protein expression (Figures 3B,C) of not only GRP78, the marker for activated ER stress pathway, but also apoptosis-related CHOP and Caspase-12 in ER stress pathway in a time-dependent manner in human cervical carcinoma cells.

### Inhibition of ER Stress Alleviated VP-Induced Apoptosis *in vitro*

**FIGURE 4:**
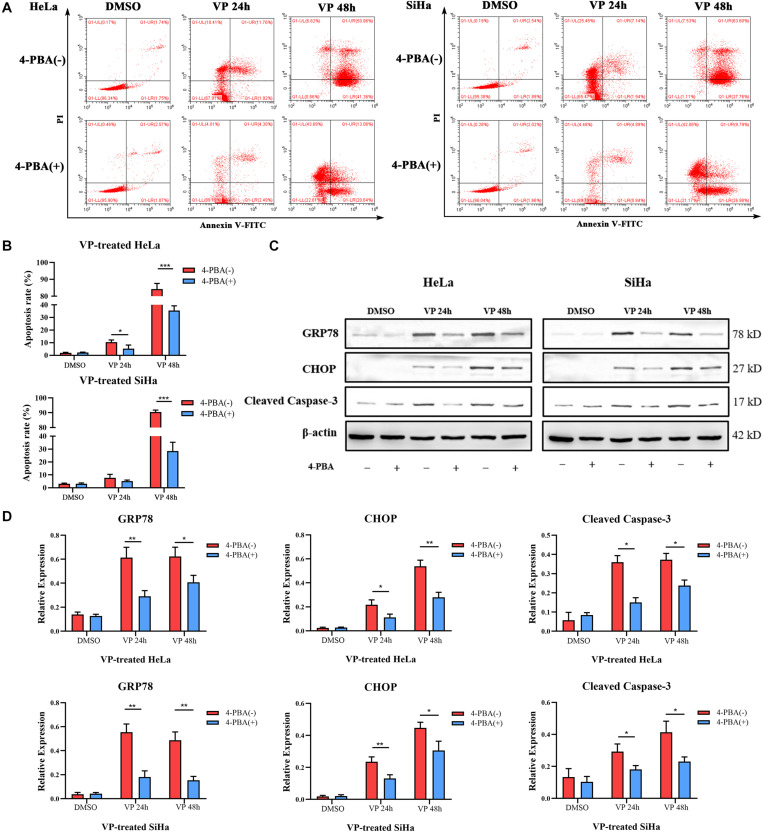
VP-induced Apoptosis can be alleviated when ER stress was inhibited *in vitro*. HeLa and SiHa cells were pre-treated with 5 mM 4-PBA or PBS (vehicle control) for 2 h before 1 μM VP administration. **(A,B)** Apoptosis was detected using flow cytometry. VP-induced apoptosis can be alleviated when cells were pre-treated with 4-PBA. **(C,D)** Protein expression of GRP78, CHOP, and Cleaved Caspase-3 was alleviated in the 4-PBA (+) groups, comparing with 4-PBA (-) groups. Data were presented as mean ± SD. Each experiment was performed for three times independently. Data were significantly different between the two linked groups (*P < 0.05; **P < 0.01, ***P < 0.001). VP = Verteporfin; 4-PBA = 4-phenyl butyric acid.

To further demonstrate VP-induced apoptosis through ER stress pathway, HeLa and SiHa cells were pre-treated with 5 mM 4-PBA or PBS (vehicle control) for 2 h before 1.0 μM VP administration. The results of Annexin V-FITC/PI flow cytometry analysis (Figures 4A,B) showed that 4-PBA efficiently alleviated VP-induced apoptosis, which was further confirmed by the decreased protein expression level of Cleaved Caspase-3 in 4-PBA-treated group (Figures 4C,D). Moreover, the protein expression levels of GRP78 and CHOP were significantly decreased in the 4-PBA-treated group, which indicated the inhibition of ER stress (Figures 4C,D).

### VP Inhibited the Growth of Tumor *in vivo* Without Impairing Ovarian Reserve

**FIGURE 5:**
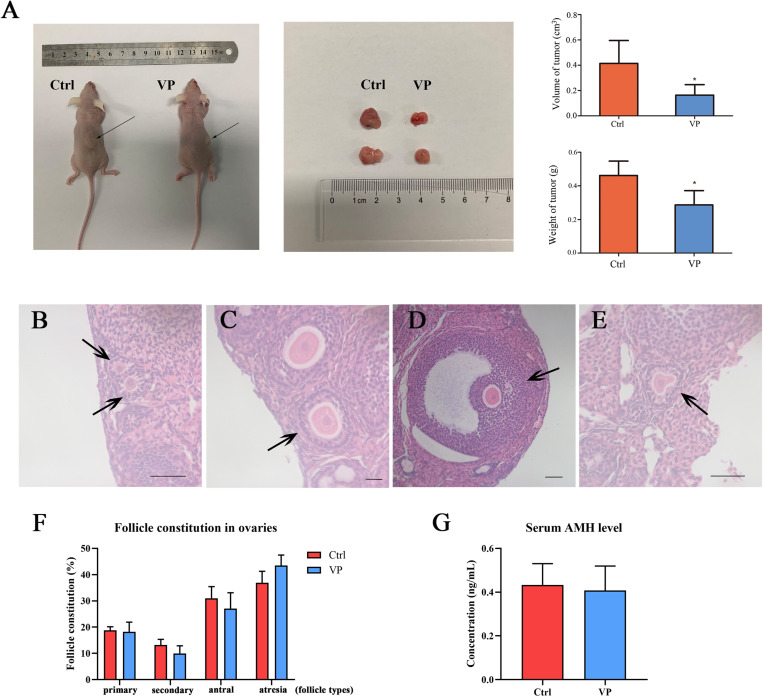
VP induced apoptosis in human cervical cancer cells *in vivo* without impairing ovarian reserve. **(A)** SiHa-based xenograft mice (*n* = 5 per group) were intraperitoneally injected with 100 mg/kg VP or normal saline (vehicle control group) every 3 day for five times when xenograft tumors (arrow) were constructed. The weight and volume of dissected tumors in the VP-treated group significantly decreased. Primary follicle [**(B)**, arrow] was exhibited as an oocyte with a single layer of cuboidal granulosa cells. Secondary follicle [**(C)**, arrow] possessed an oocyte surrounded by multi-layers of cuboidal granulosa cells without visible antrum. Antral follicle [**(D)**, arrow] was presented as a defined cumulus granulosa cell layer with a clearly antral space. The zona pellucida and oocyte in atresia follicles [**(E)**, arrow] were deformed and readily recognized. **(F)** Follicle constitution in ovaries of xenograft models was calculated, and there were no significant differences in all sorts of follicle types. **(G)** The serum AMH levels of xenograft mice were detected using ELISA, and no significant difference can be observed. Data were presented as mean ± SD. Scale bar = 50 μm. Each experiment was performed for three times independently. Data were significantly different from the control group (*P < 0.05). Ctrl, control; VP, verteporfin; AMH, anti-müllerian hormone.

To further investigate the effect of VP on human cervical carcinoma cells *in vivo*, SiHa-xenograft mice models were established. Nude mice in the VP group were intraperitoneally injected with 100 mg/kg VP every 3 days until the sacrifice day, and the animals in control group were treated with vehicle (DMSO in normal saline) of the same volume. After five doses of VP, significant retardation of tumor growth was observed *in vivo* in the VP group. The diameters and weights of dissected tumors were measured (Figure 5A), and the tumor volumes (cm^3^) and weights (g) were dramatically decreased in the VP group, compared with the vehicle group (0.41 ± 0.18 cm^3^, 0.16 ± 0.08 cm^3^, *P* = 0.023; 0.29 ± 0.09 g, 0.46 ± 0.08 g, *P* = 0.011, respectively).

The ovarian reserve was evaluated by the proportion of follicular composition and the level of serum AMH. The morphological classification of follicles in these two groups was performed and follicles in different types were exhibited in Figures 5B–E. There were no significant differences in the proportions of all sorts of follicles in the control group and VP group (Figure 5F), including primary follicle (18.82 ± 1.31% vs. 18.24 ± 4.22%, *P* = 0.774), secondary follicle (13.22 ± 2.12% vs. 10.00 ± 2.53%, *P* = 0.105), antral follicle (31.04 ± 4.39% vs. 27.06 ± 6.88%, *P* = 0.307) and atresia follicle (36.92 ± 4.34% vs. 43.47 ± 4.46%, *P* = 0.068). The serum AMH level of xenograft mice was detected using ELISA (Figure 5G). The serum AMH level in the control group was 0.433 ± 0.097 ng/mL and 0.408 ± 0.111 ng/mL for the VP group. There was no significant difference (*P* = 0.728). The concentration of AMH in each sample was all above the detection level of the ELISA kit.

## Discussion

In this study, we investigated the effect of the photosensitizer VP on human cervical carcinoma cells and elucidated its possible mechanism of action. VP was proved to suppress cell viability and induced apoptosis in human cervical carcinoma via ER stress without obvious reproductive toxicity, and may be a promising agent for cervical cancer patients with fertility desire.

HPV vaccine, which is an effective precaution against HPV infection and cervical carcinoma, decreases the incidences of this oncological disease significantly in high-income countries. However, cervical carcinoma is still the second most common cancer and the most frequent cancer inducing the death of female in 42 middle- and low-income countries (17). A great deal of attention has been paid to the fertility problems of cancer patients at child-bearing age (18), including cervical carcinoma. Although platinum-based chemotherapy strategy, like cisplatin, can alleviate the condition of cervical cancer, it still causes significant side effects (19) and accompanies with obvious reproductive toxicity and genotoxicity, such as a significant decrease and maturation arrest in germ cells (20), oocyte apoptosis in human ovary (21), amenorrhea or azoospermia (22). Therefore, it is urgently required to seek novel therapeutic strategies with lower reproductive toxicity for these patients. In this study we established xenograft models of SiHa cells, and we found that VP administration did not impair follicle constitution and serum AMH level of xenograft nude mice, which indicated that VP had no obvious reproductive toxicity and might be a promising agent for cervical carcinoma patients with fertility desire, compared with cisplatin.

Photodynamic therapy, a photosensitizer-based therapy strategy, has shown great potentiality in the treatment of various cancer types, including lung cancer (23), skin cancer (24) and so on (25). VP, a photosensitizer clinically applied to the treatment of age-related macular degeneration, has also been proved to have a potential effect on the treatment of oncological diseases without light activation (26, 27). VP is a lipoprotein-delivered benzoporphyrin derivative and can be selectively absorbed by proliferating cells such as cancer cells and neovascular endothelial cells through LDL receptors (28). This characteristic has provided VP bright prospects for application to targeted cancer treatments. In this study, we found that VP showed an outstanding suppressive effect on the viability and migration of cervical carcinoma cells through apoptosis, which was similar with the results of other studies (29). Cell apoptosis induced by VP was assessed *in vitro* (cells at both 2D and 3D levels) and *in vivo* (xenograft models). Compared with vehicle group, the apoptosis rates of cells in VP group dramatically increased in time dependency as previously reported (30). These findings provided ample evidences that VP was a promising agent for human cervical cancer.

Although VP has been tested but not yet approved as a light-based therapeutic modality for several human cancers, including fibrosarcoma-1 tumor (31), carcinoid tumor (32), and breast cancer (33). However, in these studies, the anti-tumor effects of VP must be activated by non-thermal laser at a wavelength of 693 nm, and the process of laser treatment is complicated and hard to control for non-ophthalmologists, such as gynecologists. Moreover, it is not easy to perform such a laser treatment on deep-seated tumors. The utilization of PDT against deep tumors has been greatly limited by insufficient luminous flux and the occurrence of peripheral tissue damage (34). Recently, it has been proposed that VP exerts the therapeutic effects on the malignancies not only through its light-activated destruction of neovascular vessels, but also via inducing the apoptosis or autophagy in malignant cells (35). Studies have demonstrated that, VP may still inhibit certain tumor cell lines, including ovarian cancer (36) and hepatocarcinoma (37) even without photo-activation. Therefore, in this study, we explored to attest the anti-tumor effects of VP on the cervical cancer without light activation.

No consensus has been reached on the concrete anti-tumor mechanism of VP so far. Some literatures found that the binding of free iron induced by VP can contribute to the increase of reactive oxygen species and eventually result in glioma cell deaths (38). While some other studies indicated that VP can inhibit the expression of the Yes-associated protein (YAP), a cell-growth-related molecule, to impact cell survival in ovarian cancer (36). ER stress is regarded as a crucial mechanism of many pathological process and diseases (13), and numerous studies have reported that ongoing ER stress occurred in many forms of cancer (39). In addition, it was further reported that cell apoptosis and proliferation were regulated by ER stress (40). ER chaperone GRP78, the marker of ER stress, was reported to be upregulated in cancer at levels that were in relation with disease progression (41). Prolonged activation of ER stress may initiate cell apoptosis via the overexpression of CHOP (42) as well as Caspase-12 (43), and CHOP can alter the expression of many apoptosis-related genes (44), inducing apoptosis through mitochondria-dependent pathway (45).

In this study, we found a precipitous transcriptional and translational response that the expression of GRP78, CHOP and Caspase-12 were significantly upregulated in VP group, and VP induced cell apoptosis can be alleviated when ER stress were inhibited by 4-PBA. These results indicated that VP can activate ER stress pathway and the observed apoptotic changes in VP-treated cervical cancer cells were dependent on ER stress pathway. Similar conclusion was drawn in the study exploring the relationship between ER stress and heart failure (46). Although no other researches have ever reported that VP can induce cell apoptosis in cancer via ER stress, our results suggested that the effect of ER stress in the process cannot be ignored.

There are still several limitations in this study. Firstly, the exact mechanism of VP-induced apoptosis needs to be further explored. The apoptosis rates of cervical cancer cells cannot only be reversed completely after the inhibition of ER stress pathway in our study, which indicated that ER stress may be an important but not the only pathway. Besides, we only focused on several crucial molecules involved in ER stress and apoptosis, which may be not comprehensive and convincing enough. In fact, the relationship of ER stress and apoptosis is intricate and interwoven with each other, more studies are required to reinforce our results and conclusion. In addition, in this study experiments were only carried out in cells *in vitro* and in animals, further studies about the safety of VP are required before clinical implementation for cervical cancer treatment.

## Conclusion

In summary, our study suggested that VP was able to suppress cell viability, migration and eventually induce apoptotic cell death in human cervical carcinoma via ER stress pathway without impairing ovarian reserve. This finding may provide novel insights into the promising application of VP in the treatment of cervical cancer patients with fertility desire. More comprehensive studies and in-depth analysis are required for novel VP-based approaches for cervical cancer patients.

## Data Availability Statement

The original contributions presented in the study are included in the article/supplementary material, further inquiries can be directed to the corresponding authors.

## Ethics Statement

The animal study was reviewed and approved by the Ethics Committee of Tongji Hospital, Tongji Medical College, Huazhong University of Science and Technology.

## Author Contributions

LJ, ZF, and LZ designed the research. MW, CL, YL, and QZ performed the research. MW and CL analyzed the data. MW wrote the manuscript. All authors contributed to the article and approved the submitted version.

## Conflict of Interest

The authors declare that the research was conducted in the absence of any commercial or financial relationships that could be construed as a potential conflict of interest.
